# Myocardial Hypertrophy Overrides the Angiogenic Response to Hypoxia

**DOI:** 10.1371/journal.pone.0004042

**Published:** 2008-12-29

**Authors:** Yeong-Hoon Choi, Douglas B. Cowan, Meena Nathan, Dimitrios Poutias, Christof Stamm, Pedro J. del Nido, Francis X. McGowan

**Affiliations:** 1 Department of Cardiac Surgery, Children's Hospital Boston and Harvard Medical School, Boston, Massachusetts, United States of America; 2 Department of Anesthesiology, Perioperative and Pain Medicine, Children's Hospital Boston and Harvard Medical School, Boston, Massachusetts, United States of America; University of Cincinnati, United States of America

## Abstract

**Background:**

Cyanosis and myocardial hypertrophy frequently occur in combination. Hypoxia or cyanosis can be potent inducers of angiogenesis, regulating the expression of hypoxia-inducible factors (HIF), vascular endothelial growth factors (VEGF), and VEGF receptors (VEGFR-1 and 2); in contrast, pressure overload hypertrophy is often associated with impaired pro-angiogenic signaling and decreased myocardial capillary density. We hypothesized that the physiological pro-angiogenic response to cyanosis in the hypertrophied myocardium is blunted through differential HIF and VEGF-associated signaling.

**Methods and Results:**

Newborn rabbits underwent aortic banding and, together with sham-operated littermates, were transferred into a hypoxic chamber (FiO_2_ = 0.12) at 3 weeks of age. Control banded or sham-operated rabbits were housed in normoxia. Systemic cyanosis was confirmed (hematocrit, arterial oxygen saturation, and serum erythropoietin). Myocardial tissue was assayed for low oxygen concentrations using a pimonidazole adduct. At 4 weeks of age, HIF-1α and HIF-2α protein levels, HIF-1α DNA-binding activity, and expression of VEGFR-1, VEGFR-2, and VEGF were determined in hypoxic and normoxic rabbits. At 6 weeks of age, left-ventricular capillary density was assessed by immunohistochemistry. Under normoxia, capillary density was decreased in the banded rabbits compared to non-banded littermates. As expected, non-hypertrophied hearts responded to hypoxia with increased capillary density; however, banded hypoxic rabbits demonstrated no increase in angiogenesis. This blunted pro-angiogenic response to hypoxia in the hypertrophied myocardium was associated with lower HIF-2α and VEGFR-2 levels and increased HIF-1α activity and VEGFR-1 expression. In contrast, non-hypertrophied hearts responded to hypoxia with increased HIF-2α and VEGFR-2 expression with lower VEGFR-1 expression.

**Conclusion:**

The participation of HIF-2α and VEGFR-2 appear to be required for hypoxia-stimulated myocardial angiogenesis. In infant rabbit hearts with pressure overload hypertrophy, this pro-angiogenic response to hypoxia is effectively uncoupled, apparently in part due to altered HIF-mediated signaling and VEGFR subtype expression.

## Introduction

Left-ventricular (LV) pressure-overload hypertrophy is a significant risk factor in adult and pediatric patients undergoing cardiac surgery. Hearts from these patients exhibit progressive contractile dysfunction, dilation of the ventricular wall, and increased vulnerability to ischemia-reperfusion injury as a consequence of numerous factors that include impaired glucose uptake and a reduction in high-energy phosphate concentrations [1], [2]. We have previously established that capillary density is significantly decreased in chronically hypertrophied hearts, resulting in greater diffusion distances that could act to limit the supply of energy and oxygen to this metabolically-demanding organ [3].

In children with congenital heart disease, myocardial hypertrophy is commonly associated with hypoxia and cyanosis. Under normal circumstances, tissue hypoxia induces angiogenesis through increased expression and activity of hypoxia-inducible transcription factors (HIFs) [4]. HIFs are basic helix-loop-helix (bHLH) transcription factors that increase expression of genes involved in regulating oxygen delivery capacity (*e.g.* erythropoietin and ceruloplasmin), glucose transport and metabolism (*e.g.* the glucose transporters GLUT 1–3, and hexokinase), and angiogenesis (*e.g.* VEGF and VEGFRs). Though it may assumed that the pro-angiogenic effects of tissue hypoxia would help counteract the relative capillary deficiency in the hypertrophied heart, clinical experience demonstrates that myocardial dysfunction and susceptibility to ischemia-reperfusion injury is higher in patients with combined hypertrophy and cyanosis. Therefore, we reasoned that activation of angiogenesis-related signalling pathways would be suppressed in response to hypertrophy, with resultant loss of pro-angiogenic capacity. Accordingly, we first tested the hypothesis that an increase in capillary density during hypoxia is blunted in hearts subjected to pressure-overload hypertrophy and then determined the HIF and VEGFR-subtype expression profile to identify possible points of interference.

## Materials and Methods

### Animal care

Animals received humane care in compliance with the *Principles of Laboratory Animal Care* formulated by the *National Society of Medical Research* and the *Guide for the Care and Use of Laboratory Animals* prepared by the *National Academy of Sciences* and published by the *National Institutes of Health* (NIH Publication No. 86-23, revised 1996). The Institutional Animal Care and Use Committee approved all experiments.

### Experimental Groups

Ten day old New Zealand White rabbits underwent aortic banding surgery (BAND) or sham operation (SHAM) as previously described [5], [6]. Briefly, pressure-overload hypertrophy of the left ventricle was achieved by placing 2-0 silk suture around the descending thoracic aorta, distal to the *ligamentum arteriosum*. Implantation of a fixed constriction in a rapidly growing animal induced pressure-overload ventricular hypertrophy beginning after 2–3 weeks of age. The control animals underwent the sham-procedure, including general anesthesia, lateral thoracotomy, and blunt dissection of the descending aorta. At 3 weeks of age, following recovery and weaning from mothers, the rabbit pups were transferred into a hypoxia chamber (HYPOX; Coy Laboratory Products, Grass Lake, MI, USA) or were housed in normal oxygen conditions (NORM). Each experimental group contained 7 animals. The oxygen level inside the chamber was reduced to a FiO_2_ of 0.12 by nitrogen replacement [7]–[9]. Humidity, air pressure, temperature, and CO_2_ levels were maintained at ambient levels. Food and water were provided *ad libitum*. A systemic cyanotic response was confirmed by determining hemoglobin and hematocrit levels through daily blood gas analyses using a Stat Profile 9 blood gas analyzer (Nova Biochemical, Waltham, MA).

In preliminary experiments (data not shown), protein expression and activity assays showed significant differences after 1 week of hypoxia (4 weeks post-partum), but significant changes in capillary density were not observed until week 3 (6 weeks post-partum). Subsequently, protein assays were performed at 4 weeks post-partum and capillary density was determined at 6 weeks post-partum (n = 7 at each time). At 4 or 6 weeks of age, respectively, the animals were anesthetized by intravenous injection of Ketamine/Xylazine/heparin (100 mg/kg/10 mg/kg/200 IU/kg). Hearts were rapidly excised, mounted to a modified Langendorff apparatus and perfused for 5 minutes at constant pressure (80 mm Hg) using modified Krebs-Henseleit buffer [10], [11]. Hearts were collected and processed for immunohistochemistry or used for protein isolation. All animals in the HYPOX groups were euthanized under hypoxic conditions. A separate set of animals was injected intraperitoneally 30 minutes prior to euthanasia with 100 mg/kg HypoxyProbe™-1 reagent (Millipore, Billerica, MA).

### Immunoblot Analyses and ELISA

Tissue samples from the lateral free wall of the left ventricle were obtained and processed for both total and nuclear protein as previously described [12], [13]. HIF-1 and -2, VEGF, and VEGFR-1 and -2 protein levels were determined using total tissue lysates, whereas HIF-1 DNA-binding activity was determined using nuclear extracts. SDS-PAGE and transfer to nitrocellulose were performed using standard procedures and equal protein loading of the wells was confirmed by Coomassie Brilliant Blue R250 staining of the gels. Following incubation in 5% non-fat milk suspended in Tris-buffered saline containing 0.1% Tween 20 (TBS-T), the nitrocellulose membranes were incubated with primary antibodies at 4°C overnight. For specific protein detection, the following primary monoclonal antibodies were used: anti-HIF-1α (1∶500, Calbiochem, San Diego, CA), anti-HIF-2α (1∶250, Novus Biologicals, Littleton, CO), anti-VEGFR-1 (1∶100, Millipore, Billerica, MA) and anti-VEGFR-2 (1∶100, Santa Cruz Biotechnology, Santa Cruz, CA). To determine VEGF expression levels we applied an anti-VEGF antibody, which recognizes the 121, 165, 189, and 206 isoforms (1∶2000, Millipore, Billerica, MA). Primary antibodies were detected with horseradish peroxidase-conjugated goat anti-mouse secondary antibodies (Amersham Biosciences, Piscataway, NJ) in TBS-T at the manufacturers suggested dilutions and visualized using an Amersham ECL™ kit (GE Healthcare, Piscataway, NJ). To determine erythropoietin (EPO) serum levels, blood samples were obtained from all animals immediately before euthanasia according to the manufacturer's recommendation. Following centrifugation, normalized serum fraction samples were analyzed using the Human Erythropoietin Quantikine® IVD ELISA Kit (R&D Systems, Minneapolis, MN).

### Electrophoretic Mobility Shift Assay

The DNA-binding activity of HIF-1 was determined by electrophoretic mobility shift assay[14]. Briefly, each 20 µl reaction contained 10 mM Tris-HCl (pH 7.6), 70 mM KCl, 5 mM MgCl_2_, 1 mM dithiothreitol, 1 mM EDTA (pH 8.0), 25% glycerol, 0.2% Triton X-100, 1×Complete™ protease inhibitor, 2.0 µg poly(dI-dC)·poly(dI-dC) (Amersham Biosciences), and 0.25 ng of double stranded, end-labeled HIF-1 consensus oligonucleotides (Santa Cruz Biotechnology) [15]. Some reactions contained unlabeled oligonucleotides (either identical, with mutated binding sites, or unrelated DNA of the same size). As a positive control, nuclear extracts were prepared from COS-7 cells treated with 0.15 mM cobalt chloride (CoCl_2_) for 16 hours. Probes were labeled with [γ-^32^P] ATP and polynucleotide kinase using standard techniques. Labeled DNA was purified with ProbeQuant G-50 columns (Amersham Biosciences). Densitometry of immunoblots and EMSA autoradiograms was accomplished using the NIH software Scion Image.

### Immunohistochemistry

To quantify myocardial capillary density, isolated hearts were perfused for 15 minutes with Krebs-Henseleit buffer containing 5 µM FITC-conjugated *Lycopersicon esculentum* lectin (Sigma-Aldrich, St. Louis, MO) [16]. The hearts were perfused for another 5 minutes with buffer alone and then fixed under pressure for 10 minutes with 4% paraformaldehyde (pH 7.4) (PFA). After passive fixation for 3 days in fresh PFA, heart tissue was paraffin-embedded and sectioned. Histological slides were deparaffinized, rehydrated in descending concentrations of ethanol, and incubated with 4′,6-diamidino-2-phenylindole (DAPI, Invitrogen, Carlsbad, CA,) for nuclear counterstaining. The slides were mounted with cover-slips using fluorescent mounting medium (Dako USA, Carpinteria, CA) and visualized on a Zeiss Axiovert 200 M microscope (Carl Zeiss, Thornwood, NY). Images were acquired with a Princeton Instruments MicroMAX 1300Y/S CCD camera (Roper Industries, Duluth, GA) and analyzed using MetaMorph 6.2. software (Molecular Devices, Downingtown, PA). To determine capillary density, 15 randomly selected fields of tissue cross-sections from 3 slides per animal were examined (taken from the apex, the middle and the heart base). To detect hypoxia (pO_2_<10 mmHg at tissue level), hearts were examined for pimonidazole adduct formation [17]. After sectioning, adducts were detected by immunohistochemistry, using a monoclonal antibody (1∶50) that was detected with an Alexa Fluor®488-conjugated secondary goat anti-mouse antibody (Invitrogen, Carlsbad, CA).

### Statistical analyses

Numeric data are expressed as mean±SD and statistical analyses were performed using release 15.0 of the SPSS software package (SPSS, Chicago, IL). Capillary density data were tested for significance by one-way ANOVA applying Bonferroni's correction for multiple comparisons. If normal distribution and equal variance testing was passed, Student's t-test was applied to compare individual data sets. A two-tailed probability value of less than 0.05 was considered statistically significant.

## Results

### Induction of ventricular hypertrophy

**Figure 1:**
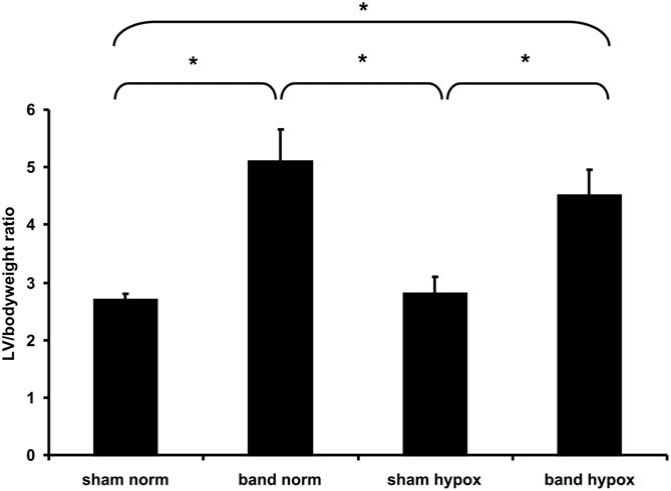
Cumulative data of the LV-to-bodyweight ratio of all experimental groups at 4 weeks of age (*p<0.01). The animals that underwent aortic banding developed significant LV hypertrophy starting at one week after surgery. Although hypoxia affected the overall growth of the animal, the development of LV hypertrophy was not significantly altered.

In preliminary experiments, hearts from each experimental group were harvested on a weekly basis, and the LV wet weight-to-body weight ratio was determined. Progression of ventricular hypertrophy was also confirmed by echocardiographic observations as previously reported [5]. Animals with aortic banding developed progressive LV hypertrophy with a peak of ‘compensated’ hypertrophy at approximately 4 weeks of age. Left ventricular dilation and failure, leading to decompensation and death, ensued at approximately 8 weeks post-partum. Our results showed no difference in the development of LV-hypertrophy between animals that were exposed to hypoxia and those subjected to normoxic conditions. Figure 1 shows the LV wet weight-to-body weight ratio at 4 weeks of age; confirming the development of significant LV hypertrophy in rabbits following aortic banding.

### Hypoxia and systemic cyanosis

**Figure 2:**
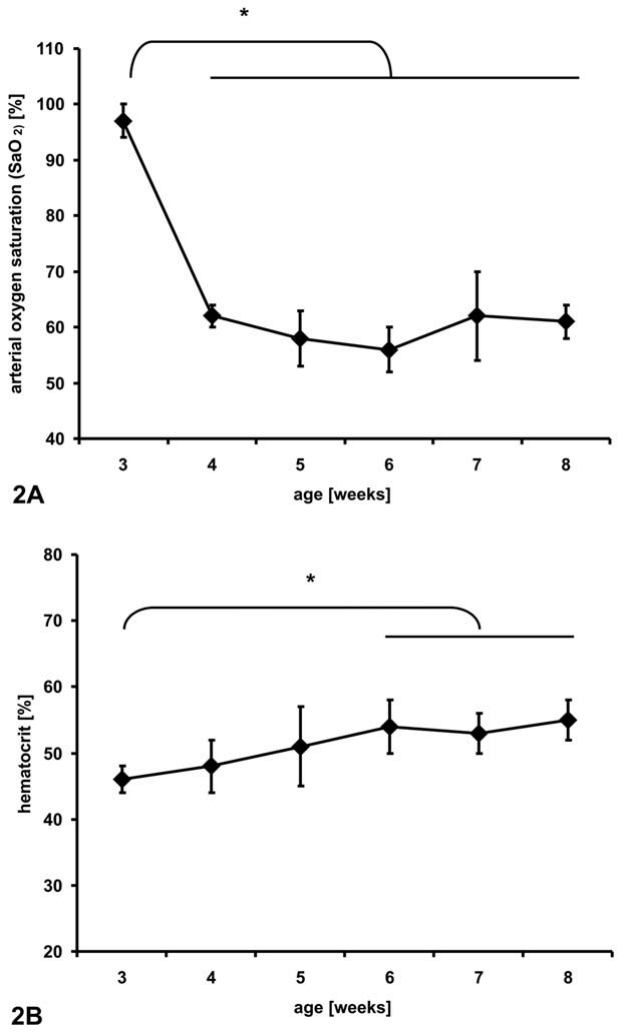
Cumulative data of blood gas analyses of sham-operated animals that have been exposed to chronic hypoxia. The shown data demonstrate immediate changes of arterial oxygen saturation upon exposure to hypoxia (FiO_2_ = 0.12) (2a) followed by increased hematocrit (2b). The banded animals responded to hypoxia in the same manner when compared to non-banded animals (not shown) (*p<0.01).

**Figure 3:**
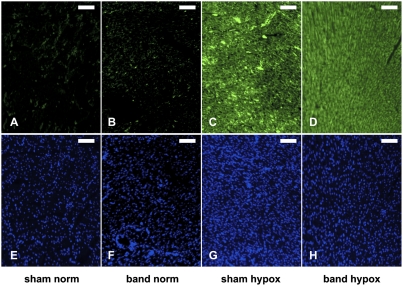
Indirect visualization of hypoxia in the myocardium. Animals from all four experimental groups were injected with 100 mg/kg pimonidazole intraperitoneally 30 minutes before euthanasia. The microscopic images show the detection of pimonidazole adducts, which form only under hypoxic conditions (pO_2_<10 mm Hg at tissue level). The top panel (A–D) shows antibody-based detection of pimonidazole adducts (green fluorescence); the bottom panel (E–H) demonstrates staining of nuclear DNA with DAPI (blue; the scale bar represents 25 µm). Animals that were exposed to chronic hypoxia showed positive staining for pimonidazole adducts, while animals that have been housed under normoxic conditions were tested negative for pimonidazole adducts. All images were acquired with identical acquisition settings (exposure time, light intensity, and neutral density filters). Surprisingly, although no hypoxia was observed in animals of the BAND NORM group, those animals showed increased expression of HIF-1α.

The FiO_2_ of 0.12 induced significant cyanosis and a typical physiological response in rabbits [9]. Daily arterial blood samples confirmed an immediate drop of the arterial oxygen saturation (SaO_2_) upon exposure to hypoxia compared with animals housed under normoxic conditions (Figure 2a). In animals that underwent aortic banding, the systemic response to hypoxia was not statistically different compared to non-banded animals (SHAM). Figure 3 shows representative immunohistochemical staining for pimonidazole adducts after exposure to hypoxia. The animals in the HYPOX groups showed positive staining with no differences between the sham operation and aortic banding groups (SHAM HYPOX and BAND HYPOX). The staining was consistent within the entire cross-section of the heart and showed no significant regional differences. Contrary to the hypothesis that pressure-overload hypertrophy would result in myocardial hypoxia, no positive pimonidazole adduct staining was detectable in animals of the BAND NORM group.

Erythropoietin (EPO) serum levels were determined by ELISA at four weeks of age. As a marker for the systemic response to reduced arterial partial oxygen pressure, animals that were exposed to chronic hypoxia showed a significant increased of EPO in the serum compared to animals under normoxia, with a consequent increase of hematocrit (Table One and Figure 2b, respectively). There was no significant difference between animals between the respective banded or sham operated groups (Table 1).

**Table 1:** Summary of serum erythropoietin concentrations in mIU/mL.

	SHAM	BAND	p
NORM	9.82±3.63	7.91±2.98	0.894
HYPOX	31.77±6.93	28.81±7.03	0.739
p	<0.001	<0.001	

Hypoxia increased EPO concentrations equally and significantly in both the sham and banded animals. Data are Mean±S.D., N = 6–8 animals/group.

### Capillary density

**Figure 4:**
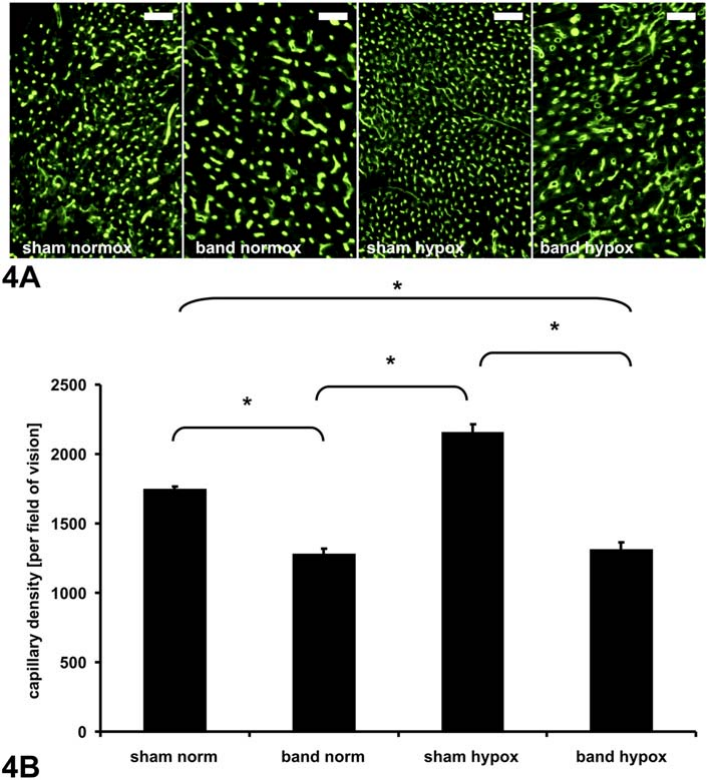
Capillary density. A) Representative micrographs of left ventricular cross-sections (6 µm thickness) showing capillary vessels in all groups (as visualized by FITC-labeled lectin, scale bar represents 100 µm). B) Vessel density quantification assessed by computer-based image analysis is shown in 4b (*p<0.01, shown as mean±SE). As a physiologic response to chronic hypoxia, sham operated animals showed a significant increase in capillary density. Animals with left ventricular hypertrophy showed a relative decrease in capillary density under normoxia. Chronic hypoxia failed to induce an increase of capillary density in hypertrophying LV myocardium.

Histological evaluation of myocardial vascular architecture displayed dense capillary distribution in the left ventricle, which was significantly increased by hypoxia in sham-operated animals. As previously observed, the capillary density was significantly reduced in animals with pressure-overload hypertrophy [16]; however, hypoxia (normally a significant activator of angiogenesis) did not induce increased vascularization in hypertrophied hearts. There was no difference in capillary density between normoxic banded animals and hypoxic banded animals (NORM BAND vs. HYPOX BAND; 1251.2±213.6 vs. 1304.3±253.4, p = 0.64), indicating a blunted angiogenic response to hypoxia. Representative photomicrographs and quantitative capillary density data, shown in Figure 4, demonstrated the pro-angiogenic effect of hypoxia in hearts of non-banded rabbits, which was negated by myocardial hypertrophy.

### HIF-1/2, VEGF, and VEGFR-1/2 expression

**Figure 5:**
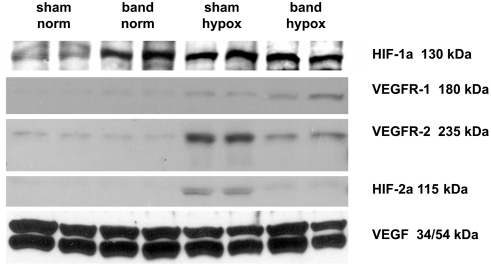
Representative immunoblots with specific detection of HIF-1α, HIF-2α in nuclear protein extracts and VEGFR-1, VEGFR-2, and VEGF in total protein extracts obtained at four weeks of age. In response to hypoxia sham operated animals showed apparent activation of HIF-1α and HIF-2α based upon increased nuclear protein concentrations, along with increased expression of VEGFR-1 and VEGFR-2. Pressure load appeared to increase HIF-1α activation and perhaps modestly increase VEGRF-1 expression, without changing HIF-2α activation. LV hypertrophy prevented hypoxia-induced increases in HIF-2α activation, decreased VEGFR-2 expression, and increased VEGFR-1 expression. VEGF levels were not affected by hypoxia or aortic banding.

HIF-1/2 are the key regulators of the hypoxic response[18]. To determine expression of those proteins, as well as their target genes VEGF and VEGFR-1/2, immunoblotting was performed (Figure 5) and densitometry data are summarized in Table 2. Under normoxic conditions, there was nearly complete suppression of HIF-1α expression in the hearts of sham-operated animals (SHAM NORM), but HIF-1α significantly increased in response to hypoxia (SHAM HYPOX). In contrast, animals with aortic banding (BAND NORM) showed elevated expression of HIF-1α under normoxia, and the elevated HIF-1α expression was not further altered by hypoxia (BAND HYPOX). HIF-2α was not detectable in sham-operated or aortic banded animals under normoxia. Under hypoxic conditions, only the animals of the SHAM HYPOX group responded with increased HIF-2α expression, whereas aortic banded animals (BAND HYPOX) showed no change in HIF-2α expression.

**Table 2:** Summary of densitometry analyses of normalized protein levels of HIF-1α, VEGFR-1, HIF-2α, VEGFR-2, and VEGF obtained by immunoblotting using lysates isolated from aortic banded and sham operated animals exposed to either normoxia or hypoxia.

	SHAM NORM	BAND NORM	SHAM HYPOX	BAND HYPOX
HIF-1α	+	+++	+++	+++
VEGFR-1	+/−	+/−	+	++
VEGFR-2	+	+	+++	+
HIF-2α	−	−	++	+/−
VEGF	+++	+++	+++	+++

Results are based upon 3–5 hearts/group.

In sham-operated normoxic hearts, very little VEGF receptor-1 protein was detected, and there was apparently no increase in VEGFR-1 protein in response to LV hypertrophy alone. VEGFR-1 expression, which can be regulated by HIF-1α and/or HIF-2α, was slightly increased under hypoxia (SHAM HYPOX). In hearts subjected to both hypoxia and hypertrophy (BAND HYPOX), increased expression of VEGFR-1 was much more pronounced. Similarly, the baseline expression of VEGFR-2 was not different in animals that underwent sham operation or aortic banding surgery under normoxia. On the other hand, a significant increase of VEGFR-2 expression under hypoxia was detected in animals of the sham-operated group. In animals with a banded thoracic aorta (BAND HYPOX), the VEGFR-2 response to hypoxia was significantly suppressed. In contrast, VEGF protein was expressed constitutively and showed no significant change in the different groups under either normoxia or hypoxia.

### HIF-1α DNA-binding activity

**Figure 6:**
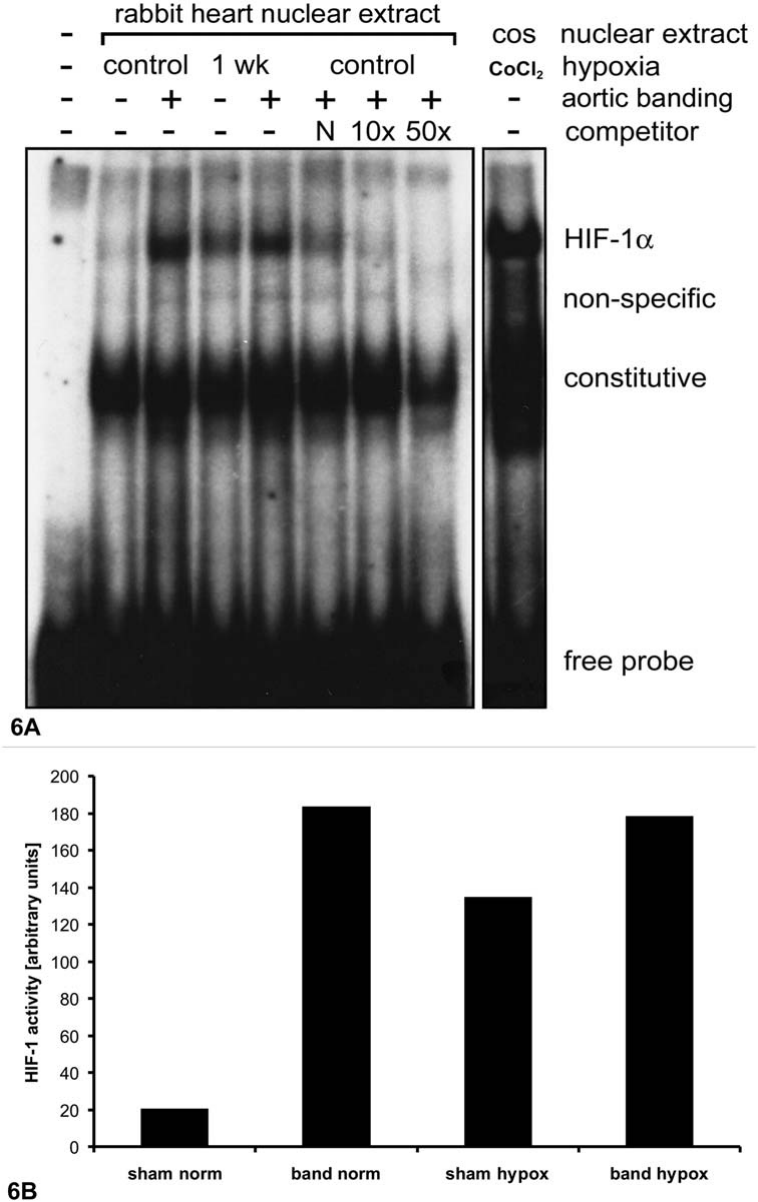
A) Representative autoradiograph of electrophoretic shift assay (EMSA) of specific binding of HIF-1α nuclear protein. Increased activity of HIF-1α was detected in hypertrophic myocardium under normoxic conditions with further elevation under hypoxia. As a positive HIF-1α control, nuclear extracts were obtained from COS-7 (COS) cells treated with 0.15 mM CoCl_2_ for 16 hours and subjected to parallel EMSAs. B) Densitometry analysis of HIF-1α EMSAs.

To determine the activity of HIF-1α, nuclear protein was analyzed by the electrophoretic mobility shift assay [15]. HIF-1α DNA-binding activity paralleled the protein expression data described above. There was very little specific binding to labeled HIF-1α consensus sequences in sham-operated animals under normoxia. Upon exposure to hypoxia, nuclear HIF-1α DNA-binding was significantly increased. In aortic banded animals, binding to the HIF-1 consensus sequence was elevated under normoxic conditions, and further increased as a result of exposure to hypoxia (Figure 6).

## Discussion

Our principal finding is that the normal pro-angiogenic response to hypoxia seen in non-hypertrophied hearts fails to occur in left ventricular myocardial hypertrophy. While capillary density increases in response to hypoxia in non-hypertrophied hearts, it remains unchanged in hearts that have a pressure-overload hypertrophic response superimposed on hypoxia. This likely renders the hypertrophied heart more susceptible to ischemia-reperfusion injury and potentially contributes to cardiomyocyte dysfunction and ventricular failure. At the molecular level, it appears that hypoxia-induced HIF-2α and VEGFR-2 expression are reduced in left ventricular hypertrophy, and that these are necessary, with or without the participation of HIF-1α, to promote angiogenesis in response to hypoxia. Conversely, hypoxia-mediated induction and activation of HIF-1α and VEGFR-1 are preserved during hypertrophy, which results in increased expression of VEGFR-1 in hearts that are both hypoxic and hypertrophied. Taken together, the hypoxia-induced angiogenic response in is uncoupled in hypertrophied hearts, presumably due to differential effects of HIF and VEGFR signaling.

Differential expression of isoforms of the transcription factor HIF and the subsequent induction of target genes such as VEGFR-1 and VEGFR-2 appear responsible for the response to hypoxia in hypertrophied hearts. Within the HIF family, three protein subtypes have been described, of which HIF-1α and HIF-2α have been characterized in the greatest detail [19]. HIF is a heterodimer composed of an alpha-(HIF-α) and beta-subunit (HIF-β). While HIF-β is expressed constitutively, expression of HIF-α is directly regulated by low tissue pO_2_. Under normoxic conditions, HIF-α is the target of hydroxylation by prolyl hydroxylases. The hydroxylated protein is then ubiquitinated and degradation by the proteosome. Under hypoxia, HIF-α stabilizes and translocates to the nucleus as a heterodimer with the β-subunit and activates gene transcription. In the present experiments, hypertrophy increased HIF-1α expression and activity without evidence of tissue hypoxia. This result is consistent with evidence that HIF-1α expression and activation can be promoted by non-hypoxic mechanisms likely to be active in hypertrophying hearts, including increased reactive oxygen species production, mechanical stretch-activated channels, and phosphatidylinositol 3-kinase (PI3-K)-dependent Akt phosphorylation [20].

The relative roles of the different HIF moieties remain unclear. Independent, complementary, redundant, and opposing functions have all been described for HIF-1α and HIF-2α, depending upon developmental stage, experimental conditions, activating stimulus, and cell type [21]–[25]. Similarly, it appears that expression of VEGFR-1 and VEGFR-2 can be primarily regulated by HIF-1α, HIF-2α, or at times both [26]–[30]. Within the VEGF receptor family, VEGFR-2 is better characterized and plays an important role in induction of angiogenesis. Reduced expression of VEGFR-2 in hypertrophied myocardium is one possible explanation of decreased capillary density in these hearts. As the systemic response to hypoxia in animals that underwent aortic banding appears to be intact, it seems likely that alteration in myocardial gene expression and protein content in response to pressure overload results in inhibition of HIF-2 and VEGFR-2 expression and, thus, capillary growth.

Increased expression of VEGFR-1 by HIF-1α has been well established, but the consequence of activation of this signalling pathway and its role in stimulating VEGFR-2 expression is not as well characterized [31], [32]. VEGFR-1 is expressed in two forms (both of which are detectable by the antibody used in our experiments). These include the full-length membrane bound receptor, capable of transducing a cellular signal, and a soluble receptor, capable of sequestering ligand or dimerizing with the full-length receptor to prevent signal transduction [33], [34]. It is known that the extracellular portion and the soluble fraction of VEGFR-1 have a 10-fold higher affinity for the VEGF ligand with limited or no detectable autophosphorylation activity. Furthermore, VEGFR-2 is phosphorylated approximately 10-fold more efficiently upon ligand binding [32]. Seetharam *et al.* demonstrated a high binding affinity of VEGFR-1 to VEGF without generating a mitogenic response in transfected NIH3T3 fibroblasts that over-expressed VEGFR-1 [35]. As a consequence, VEGFR-1 has been referred to by some as an impaired protein kinase that can function as a “decoy” receptor [36], [37], particularly in its soluble form. Based on this information, excessive expression of VEGFR-1 could result in increased binding to VEGF ligand, resulting in competitive suppression of VEFGR-2 activation and the angiogenic response. Hiratsuka *et al.* reported that VEGFR-1 is essential for normal development of the vasculature [38]. They showed that mice expressing a truncated form of VEGFR-1 die at embryonic day 8.5, whereas mice lacking only the VEGFR-1 tyrosine kinase domain survived and showed normal development of the vasculature [38]. Therefore, based on the increased binding affinity and decreased signaling capability, VEGFR-1 does appear to have the ability to act as a “decoy” receptor as well as an angiogenesis-inducing receptor [39]. As a result, the marked increase in HIF-1α and VEGFR-1 expression in the hypertrophied myocardium, in combination with the attenuated HIF-2α and VEGFR-2 response, most likely contribute to the observed lack of angiogenic response to hypoxia in our studies. Interestingly, Ahmad *et al.* showed that elevated expression of soluble VEGFR-1 in the inhibits placental angiogenesis [40], a result that is consistent with our observations.

### Limitations of the study

Even though we believe that our experimental design was appropriate to test the primary hypothesis (*i.e.* that the pro-angiogenic response to hypoxia would be blunted in hypertrophied hearts), the conclusion that differential VEFGR-subtype expression and activity might be the cause for this effect are based upon association and the current state of knowledge. To substantiate the cause-effect relationship, future experiments using VEGFR-1 knock-out mice or siRNA approaches to selectively suppress VEGFR-1 expression in wild type animals are necessary. The cellular source(s) of the various HIF and VEGF subtypes were also not determined by the present experiments; detailed immunohistochemical studies and *in vitro* experiments will be necessary to distinguish the relative contributions from cardiomyocytes, endothelial cells, fibroblasts, and smooth muscle. Other mechanisms of angiogenesis inhibition, occurring at different stages of hypertrophy, may also be involved. For example, accumulation of p53 has recently been shown inhibit HIF-1α activity and thereby hinder angiogenesis during the later stages of severe pressure overload hypertrophy progressing to contractile failure [41].

### Conclusion

Our studies provide evidence that pressure overload hypertrophy is associated with decreased capillary density, increased expression of HIF-1α, and the potential decoy VEGF receptor, VEGFR-1, and decreased expression of HIF-2α and VEGFR-2. Unlike the non-hypertrophied heart, hypoxia further increased VEGF-1 expression without a concomitant increase in the pro-angiogenic receptor, VEGFR-2. These changes occurred relatively early during the development and progression of hypertrophy. It appears that HIF-2α signaling is necessary to mount a myocardial angiogenic response. This differential regulation of HIF-1α and HIF-2α in pressure overload hypertrophy may explain the lack of angiogenesis as muscle mass increases and help determine the eventual development of contractile dysfunction and ventricular dilation.
